# Prognostic Role of Lymphocyte-to-C-Reactive Protein Ratio in Patients with Pulmonary Arterial Hypertension

**DOI:** 10.3390/jcm13247855

**Published:** 2024-12-23

**Authors:** Meng-Qi Chen, Chuan-Xue Wan, Jun Tong, An Wang, Bin-Qian Ruan, Jie-Yan Shen

**Affiliations:** Department of Cardiology, Renji Hospital, School of Medicine, Shanghai Jiao Tong University, 160 Pujian Road, Shanghai 200127, China

**Keywords:** pulmonary arterial hypertension, lymphocyte-to-C-reactive protein ratio, inflammation, nomogram, prognosis

## Abstract

**Background:** Inflammation plays a critical role in the prognosis of patients with pulmonary arterial hypertension (PAH). The lymphocyte-to-C-reactive protein ratio (LCR), as a novel inflammatory marker, has not been studied in patients with PAH. The objective of this study was to investigate the prognostic value of the LCR in patients with PAH. **Methods:** A retrospective cohort study was conducted on 116 patients with PAH diagnosed in Renji Hospital, School of Medicine, Shanghai Jiao Tong University, from January 2014 to December 2018. The primary outcome was a composite endpoint that included lung transplantation, rehospitalization for PAH, and all-cause death. The LCR is the ratio of the blood lymphocyte count to the C-reactive protein concentration. **Results:** A total of 116 patients with PAH were included in this study, with an average age of 41.53 years; 92.2% were female, and the event rate was 57.8%. Restricted cubic spline analysis confirmed a linear association between the LCR and the risk of clinical worsening events. Multivariate Cox proportional hazards analysis showed that the LCR was significantly negatively associated with clinical worsening events, with hazard ratios and 95% confidence intervals of 0.772 (0.614–0.970). The Kaplan–Meier curve showed that event-free survival decreased significantly when the LCR was less than 1.477. LASSO regression selected four potential predictors, including the LCR, to construct a nomogram. The nomogram had a high predictive strength, with an area under the ROC curve of 0.805 (0.713–0.896). The calibration curves and decision curve analysis indicated that the nomogram had good predictive performance and the ability to guide clinical management. **Conclusions:** The LCR is a valuable prognostic marker for predicting long-term clinical events in patients with PAH, and the nomogram incorporating the LCR could effectively stratify risk and guide clinical decision making.

## 1. Introduction

Pulmonary arterial hypertension (PAH) is a progressively debilitating condition characterized by obstructive remodeling of the pulmonary arteries [1]. As the first group of pulmonary hypertension to be characterized, PAH includes subtypes, such as idiopathic PAH (IPAH), hereditary PAH, and disease-associated PAH, with disease-associated PAH, including connective tissue disease-associated PAH (CTD-PAH), congenital heart disease-associated PAH (CHD-PAH), and portopulmonary hypertension (PoPH) [2]. Although symptoms and survival vary among different subtypes, the role of inflammation, particularly of lymphocytes and C-reactive protein (CRP), is critical in the pathogenesis and prognostic survival of PAH in various subgroups.

Inflammation, as an essential component of innate immunity, is an important driver of the pathogenesis and progression of PAH [3]. Inflammatory cell infiltrates, such as macrophages, lymphocytes, and monocytes, can be seen in the pulmonary arterial walls of patients with PAH [3,4]. Cytokines (i.e., interleukin-1β, interleukin-6, and tumor necrosis factor-alpha) released by these inflammatory cells can stimulate smooth muscle cell proliferation, endothelial cell damage, and vascular endothelial dysfunction, thereby promoting vascular remodeling and increasing pulmonary vascular resistance (PVR) [3,4,5,6]. Various inflammatory markers, such as CRP and the neutrophil-to-lymphocyte ratio, are correlated with disease severity and prognosis in patients with PAH [7,8]. In addition, lymphocytes, particularly CD4+ T cells, are important risk factors for PAH disease progression [9,10]. Recently, the lymphocyte-to-C-reactive protein ratio (LCR) has emerged as a new inflammatory marker with potential prognostic value in various inflammation-related diseases [11,12,13]. A retrospective multicenter cohort study indicated that the LCR was an independent prognostic biomarker for hemodialysis patients [13]. The LCR is the ratio of the blood lymphocyte count to the CRP concentration, reflecting the immune response and systemic inflammation. Therefore, monitoring lymphocyte changes and CRP levels over time can also provide valuable information about the effectiveness of therapeutic interventions and disease progression.

Although both lymphocytes and CRP are risk factors for PAH, the role of the LCR in the prognosis and risk stratification of PAH remains unclear. Hence, we aimed to explore the prognostic role of the LCR in patients with PAH, as well as its function in risk stratification of PAH. Incorporating the LCR into clinical assessment models may help to identify patients at higher risk of disease progression and optimize patient management strategies.

## 2. Materials and Methods

### 2.1. Study Population

This retrospective cohort study was based on data from Renji Hospital, School of Medicine, Shanghai Jiao Tong University, between January 2014 and December 2018, including 116 patients with PAH. The inclusion criteria for patients were (1) patients aged ≥18 years; (2) patients diagnosed with PAH confirmed by right heart catheterization (RHC); (3) patients with cardiac color Doppler ultrasound and laboratory examination data within 1 month before and after RHC. The hemodynamic criteria for PAH were a mean pulmonary artery pressure (mPAP) ≥ 25 mmHg, PVR ≥ 3 Wood units, and pulmonary artery wedge pressure (PAWP) ≤ 15 mmHg. The exclusion criteria were (1) patients with a history of malignant tumors, lung diseases, and severe heart failure; (2) patients with a history of mental illness; (3) patients with a history of inflammatory infections. This study was approved by the Ethics Committee of Renji Hospital, School of Medicine, Shanghai Jiao Tong University, China (Ethic number: KY2022-037-B). All patients in this study provided informed consent.

### 2.2. Data Collection

This study collected the survival status of patients who were diagnosed with PAH and underwent outpatient visits, rehospitalizations, or telephone interviews for five years after RHC. This study collected relevant information from the patients, including their demographics, WHO functional class (WHO-FC), 6 min walk distance (6MWD), PAH initial therapy, blood indicators, RHC data, and echocardiography. The demographic information included gender, age, height, weight, and other data. PAH initial therapy included monotherapy, dual therapy, and triple therapy with targeted medications for patients with PAH. The blood indicators included B-type natriuretic peptide (BNP), CRP, and blood cell examination, including white blood cells, lymphocytes, monocytes, neutrophils, and platelet count. The blood indicators were assessed before the patient’s first hospitalization for right heart catheterization. The echocardiography data mainly included the right ventricular diameter, tricuspid annular systolic excursion (TAPSE), and estimated pulmonary artery systolic pressure (PASP) data. The RHC data included the right atrial pressure (RAP), mPAP, PAWP, cardiac output (CO), cardiac index, PVR, mixed venous oxygen saturation (SvO_2_), and other indicators.

### 2.3. Definition

The body mass index (BMI) was defined as the weight in kilograms divided by the square of the height in meters (kg/m^2^). The cardiac index was calculated by dividing cardiac output by body surface area. The PVR was calculated based on the data of the mPAP, PAWP, and CO, using the formula PVR = (mPAP − PAWP)/CO × 80. The 6 min walk distance was measured as the distance that a patient could walk quickly in a straight line on a flat, spacious surface for six minutes. The lymphocyte-to-C-reactive protein ratio was defined as the ratio of the blood lymphocyte count to the CRP concentration. The composite endpoint of this study was the initial occurrence of clinical worsening events, including all-cause death, lung transplantation, and rehospitalization for PAH. The follow-up time was defined as the time from RHC to the occurrence of clinical worsening events.

### 2.4. Statistical Analysis

Categorical variables were presented as frequencies (percentages), while continuous variables were presented as mean (standard deviation) or median (interquartile range), depending on the distribution of the data. Multiple imputation was used to complete missing covariate data in this study. For missing at random data, multiple imputation handles missing data by creating multiple complete datasets, then analyzes these imputed datasets and combines the results to more accurately reflect the overall characteristics [14]. Based on Cox proportional hazards model, restricted cubic spline analysis was used to analyze the linear relationship between the inflammatory marker of the LCR and the risk of clinical worsening events in patients with PAH. Multivariable Cox proportional hazards analysis was used to evaluate the relationship between the LCR and clinical worsening events in patients with PAH. The optimal cut-off value of the LCR was determined using the optimal *p*-value method, and then Kaplan–Meier method was used to generate survival curves based on the categorical LCR. The log-rank test was used to detect the differences between the higher LCR group and the lower LCR group. LASSO regression analysis was used for feature selection of clinical and inflammatory variables. Lasso regression is a linear regression technique that adds an L1 regularization term to the loss function. This L1 regularization works by imposing a penalty on the absolute values of the model coefficients, reducing less important feature coefficients to zero. This process not only simplifies the model by effectively selecting a relevant subset of features but also enhances model interpretability and helps prevent overfitting. After feature selection, a nomogram was created to integrate multiple clinical features and the LCR, visualizing the relationship between the variables and predicting the probability of clinical worsening events in patients with PAH. The receiver operating characteristic (ROC) curve, calibration curve, and decision curve analysis (DCA) were used to evaluate the predictive ability of the model for clinical worsening events in patients with PAH. All data analyses were performed using R software (version 4.4.1; R Foundation for Statistical Computing, Vienna, Austria) and SPSS 27.0 software (IBM Corp, Chicago, IL, USA), with a *p*-value < 0.05 considered statistically significant.

## 3. Results

### 3.1. Baseline Characteristics of Patients with PAH

A total of 116 patients with PAH aged ≥ 18 years were included in this study, including 28 patients with IPAH, 66 patients with CTD-PAH, 17 patients with CHD-PAH, and 5 patients with PoPH. Table 1 shows the baseline characteristics of patients with PAH. The mean age of the 116 patients with PAH was 41.53 (13.33) years, and the female proportion was 92.2%. The proportion of WHO functional class III/IV in patients with PAH was 31.9%. Among the total patients with PAH, the mean BMI was 21.60 (3.40) kg/m^2^, the mean 6MWD was 382.49 (121.58) meters, and the mean BNP level was 359.19 (547.08) pg/mL. Among the baseline variables in patients with PAH, the 6MWD, WHO-FC, CRP, platelet count, and LCR showed statistical differences between the two groups with and without clinical worsening events, while other variables (including age, female, height, weight, BMI, PAH subtype, initial therapy, seven variables in RHC, four variables in echocardiography, WBC, lymphocyte, monocyte, platelet, and BNP) did not show statistical differences.

### 3.2. Association Between the LCR and PAH Prognosis

**Figure 1:**
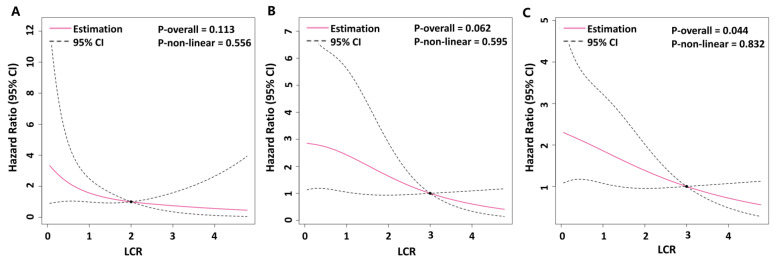
Restricted cubic spline analysis of LCR and risk of clinical worsening events in patients with PAH. Changes in hazard ratios for 1-year (**A**), 3-year (**B**), and 5-year (**C**) clinical worsening events across different baseline levels of the LCR. Hazard ratios and 95% confident intervals were estimated using Cox proportional hazards models. LCR, lymphocyte-to-C-reactive protein ratio; PAH, pulmonary arterial hypertension.

In Figure 1, the dose–response relationship between the LCR and the risk of clinical worsening events was evaluated using restricted cubic spline analysis based on a Cox proportional hazards model. A linear association between the LCR and the risk of clinical worsening events was observed over the five-year period (*p* for overall = 0.044; Figure 1C). No non-linear associations were detected for the one-year (*p* for non-linearity = 0.556; Figure 1A), three-year (*p* for non-linearity = 0.595; Figure 1B), and five-year (*p* for non-linearity = 0.832) assessments.

To determine the optimal cut-off value of the LCR for the risk of clinical worsening events in patients with PAH, the optimal *p*-value method was used, and the estimated cut-off value of the LCR was 1.477. Table 2 presents the results of the Cox proportional hazards model between the LCR and clinical worsening events. For the one-year clinical worsening events endpoint, the LCR was not significantly associated with the risk of clinical worsening events in patients with PAH, whether analyzed as a continuous or categorical variable. In model 1, without adjustment for variables, the LCR showed a significant association with the risk of clinical worsening events in patients with PAH, with a hazard ratio (HR) and 95% confident interval (CI) of 0.688 (0.515–0.918) for the continuous LCR and 0.283 (0.121–0.660) for the categorical LCR at the three-year follow-up, with an HR and 95% CI of 0.744 (0.594–0.933) for the continuous LCR and 0.343 (0.169–0.693) for the categorical LCR at the five-year follow-up. After adjusting for age, gender, and WHO-FC, the associations remained statistically significant, with an HR and 95% CI of 0.708 (0.531–0.944) for the continuous LCR and 0.299 (0.128–0.698) for the categorical LCR at the three-year follow-up, with an HR and 95% CI of 0.760 (0.606–0.954) for the continuous LCR and 0.356 (0.176–0.722) for the categorical LCR at the five-year follow-up. After further adjustment for BMI, 6MWD, PAH subtype, PAH initial therapy, BNP, neutrophil count, monocyte count, RAP, mPAP, PAWP, PVR, SvO_2_, and TAPSE/PASP, the HRs and 95% CIs for the continuous LCR were 0.708 (0.531–0.944) at the three-year and 0.772 (0.614–0.970) at the five-year follow-up, and the categorical LCRs were 0.299 (0.128–0.698) at three years and 0.361 (0.178–0.732) at five years.

**Figure 2:**
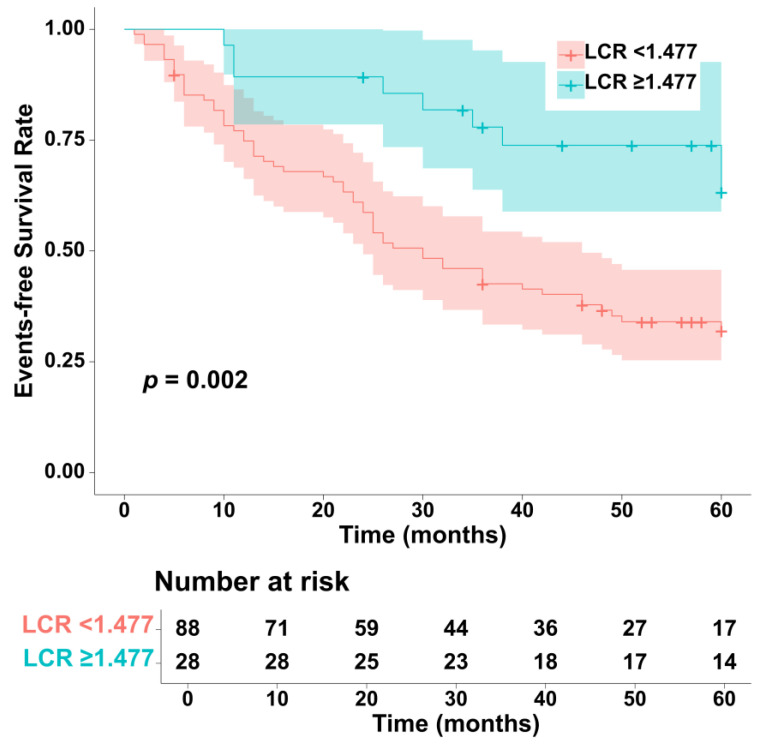
Kaplan–Meier survival analysis of LCR and the risk of clinical worsening events in patients with PAH. The LCR was expressed as a categorical variable according to optimal *p*-value method: LCR < 1.477 and LCR ≥ 1.477. Differences between the two groups were compared using the log-rank test. LCR, lymphocyte-to-C-reactive protein ratio; PAH, pulmonary arterial hypertension.

The one-year, three-year, and five-year occurrence rates of clinical worsening events in patients with PAH in this study were 22.4%, 48.3%, and 57.8%, respectively. The results of the Kaplan–Meier survival curve indicate that categorical LCR had a good predictive ability for the prognosis of patients with PAH (Figure 2). It can be seen that the lower LCR group (LCR < 1.477) had a significantly decreased event-free survival rate compared to the higher LCR group (LCR ≥ 1.477), with a log-rank *p*-value of 0.002.

### 3.3. Construction and Evaluation of Prognosis Model

**Figure 3:**
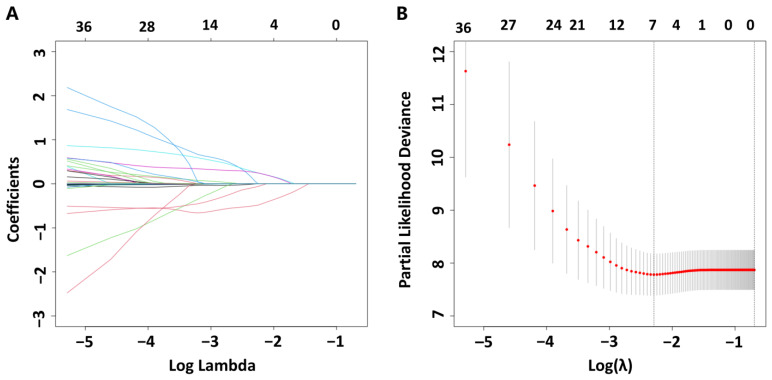
Selection of potential predictors of LASSO regression in patients with PAH. (**A**) The coefficient shrinkage process for 28 variables, with colored lines representing the changes in coefficients of different features at various levels of shrinkage. (**B**) A 10-fold cross-validation to determine the optimal penalty parameter lambda. A vertical line is drawn at the point of 1 standard error (1-SE) of the minimum criterion. LASSO, least absolute shrinkage and selection operator; PAH, pulmonary arterial hypertension.

Based on the baseline demographics, RHC parameters, echocardiographic variables, and laboratory parameters of patients with PAH, 28 variables were included in the coefficient shrinkage process of LASSO regression (Figure 3A). Ten-fold cross-validation was conducted to select relevant variables based on the lambda.1se criterion. When the optimal lambda.1se was 0.500, four prognostic factors (the LCR, PAH subtype, 6MWD, and WHO-FC) with nonzero coefficients were selected (Figure 3B).

**Figure 4:**
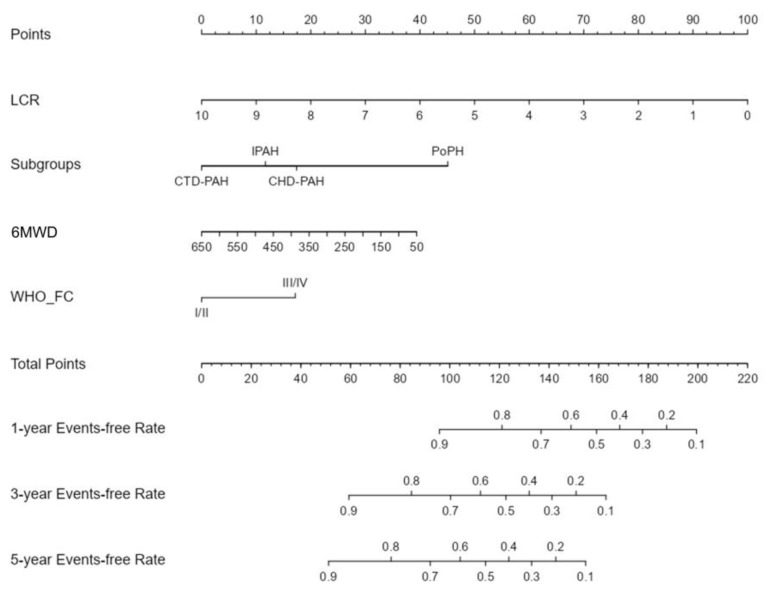
Nomogram for clinical worsening events prediction in patients with PAH. The nomogram was based on Cox proportional hazards models, integrating four variables: lymphocyte-to-C-reactive protein ratio (LCR), PAH subtype (subgroups), 6 min walk distance (6MWD), and WHO cardiac functional class (WHO-FC). PAH, pulmonary arterial hypertension.

Based on the four selected variables, we constructed a nomogram that incorporated the LCR, PAH subtype, 6MWD, and WHO-FC to predict the 1-year, 3-year, and 5-year event-free survival rates in patients with PAH (Figure 4). Patients with higher total points had a lower probability of event-free survival, indicating an increased risk of clinical worsening events. The nomogram assigned a point value based on the alignment of each variable with the top “Points” bar. For a given PAH patient, the scores allocated based on the LCR, PAH subtype, 6MWD, and WHO-FC were then summed up. This value was subsequently recorded on the “Total Points” bar below the variables and was used to calculate the risk of clinical worsening events at 1 year, 3 years, and 5 years based on the “Events-free Rate” bar at the bottom. For example, a CTD-PAH patient with an LCR of 0.371, a 6MWD of 310 m, and classified as WHO-FC I/II had estimated probabilities of event-free survival of 81.3% at 1 year, 54.1% at 3 years, and 40.3% at 5 years.

**Figure 5:**
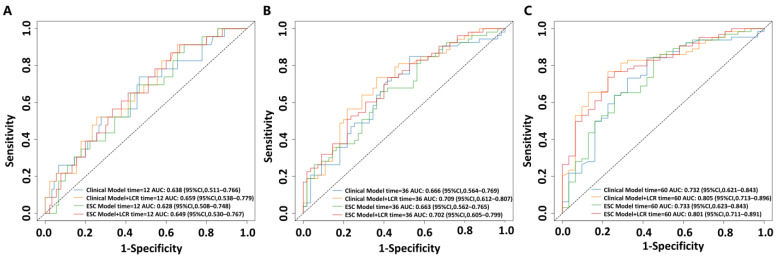
Receiver operating characteristic curves of four models for 1-year (**A**), 3-year (**B**) and 5-year (**C**) clinical worsening events. The dotted line represents the reference line. Clinical model included variables of PAH subtype, 6 min walk distance, and WHO cardiac functional class. European Society of Cardiology (ESC) model included variables of B-type natriuretic peptide, 6 min walk distance, and WHO cardiac functional class.

To assess the predictive performance of the nomogram, the ROC curves were plotted for the clinical model (including PAH subtype, 6MWD, and WHO-FC) and the nomogram model for the prediction of clinical worsening events, as well as the 2022 ESC/ERS model (including BNP, 6MWD, and WHO-FC) and the ESC model plus the LCR (Figure 5). The one-year, three-year, and five-year areas under the ROC curve (AUC) for the clinical model were 0.638 (0.511–0.766), 0.666 (0.564–0.769), and 0.732 (0.621–0.843), respectively. For the nomogram, the one-year, three-year, and five-year AUCs were 0.659 (0.538, 0.779), 0.709 (0.612–0.807), and 0.805 (0.713–0.896), respectively. The AUCs for the ESC model at one year, three years, and five years were 0.628 (0.508–0.748), 0.663 (0.562–0.765), and 0.733 (0.623–0.843), respectively. When combining the ESC model with the LCR, the one-year, three-year, and five-year AUCs were 0.649 (0.530–0.767), 0.702 (0.605–0.799), and 0.801 (0.711–0.891), respectively. These results indicate that incorporating the LCR into both the clinical model and the ESC model significantly enhanced the predictive capability of the models.

**Figure 6:**
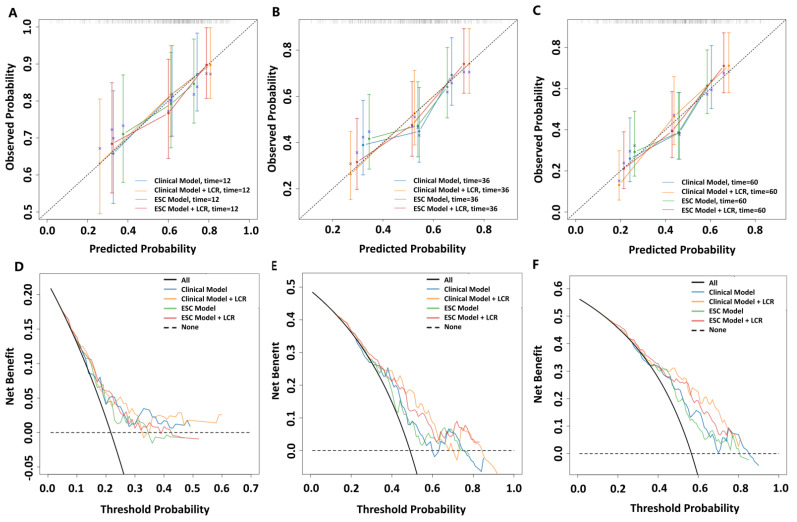
Validation of the four models through calibration curve and decision curve analysis. (**A**–**C**) Calibration curves of the four models for 1-year (**A**), 3-year (**B**), and 5-year (**C**) clinical worsening events, with the x-axes representing actual event probabilities and the y-axes representing predicted event probabilities. The dotted line represents the ideal prediction. (**D**–**F**) Decision curve analysis of the four models for 1-year (**D**), 3-year (**E**), and 5-year (**F**) clinical worsening events. Clinical model includes variables of PAH subtype, 6 min walk distance, and WHO cardiac functional class. European Society of Cardiology (ESC) model includes variables of B-type natriuretic peptide, 6 min walk distance, and WHO cardiac functional class.

To assess the differences between the predicted event rates and the actual event rates of the four models at one, three, and five years, the calibration curves were plotted to evaluate the predictive ability of the four models (Figure 6A–C). The calibration curves demonstrated that both the clinical model and the nomogram performed better than the ESC model and the ESC model plus the LCR during the one-year follow-up. At the three-year and five-year follow-ups, the nomogram model exhibited the best performance among the four models. Furthermore, to better fit the clinical needs, we used the decision curve analysis to compare the clinical benefits of the four models in predicting clinical worsening events in patients with PAH (Figure 6D–F). Consistent with the results of the calibration curve, in the one-year prediction model, both the clinical model and the nomogram had higher clinical benefits than the ESC model and the ESC model plus the LCR. In the three-year prediction model, the nomogram had the greatest clinical benefits among the four models at threshold probabilities ranging from 48% to 64%. Similarly, in the five-year prediction model, the nomogram model based on the LCR had the greatest clinical benefits at threshold probabilities between 58% and 76%, indicating that the nomogram model provided the greatest advantage in improving patient prognosis and guiding clinical decision making.

## 4. Discussion

In this retrospective cohort study, we seem to be the first to explore the relationship between the inflammatory marker of the LCR and the risk of clinical deterioration in patients with PAH. Our study suggests that the LCR could serve as a valuable prognostic marker for assessing the risk of clinical worsening in patients with PAH, indicating its potential utility in clinical management.

The prognostic value of the LCR was assessed using a variety of statistical analyses among patients with PAH in a comprehensive study. Using multivariable Cox proportional hazards analysis and Kaplan–Meier survival curves, our study found that a lower LCR was significantly associated with a higher risk of clinical deterioration events in patients with PAH. The restricted cubic spline analysis corroborated a linear correlation between the LCR and the risk of clinical outcomes. According to the results of the ROC curves, calibration curves, and decision curve analysis, the nomogram based on the LCR demonstrated the best predictive performance among all models. Importantly, whether the LCR was added to the clinical model or the ESC model, the predictive ability of the models has been significantly improved.

Inflammation may significantly influence the prognostic progression of PAH, and our research offers new evidence to support this. Previous research has primarily concentrated on other inflammatory markers to predict clinical deterioration in patients with PAH [15,16]. A French study reported that inflammatory biomarker panels of β-NGF, TRAIL, and CXCL9 were significantly associated with the prognosis of PAH [17]. A study on a UK cohort reported that the circulating markers of inflammation and angiogenesis were independently associated with the five-year survival of patients with PAH [18]. Additionally, a population study conducted in China suggested that the systemic inflammatory response index was a reliable predictor of prognosis in patients with IPAH [15]. However, the novel inflammatory marker of the LCR has not been extensively studied in this context. Consistent with the abovementioned studies, we observed that the LCR was a strong prognostic predictor for patients with PAH.

The increase in lymphocytes and CRP levels was significantly correlated with the prognosis of PAH [7,9]. Lymphocytes are a specific type of immune cell that play a paramount role in the inflammatory response associated with PAH [19]. Studies have shown an increased presence of lymphocytes, especially T cells, in the pulmonary arteries of patients with PAH [20]. These infiltrating lymphocytes release various pro-inflammatory cytokines (e.g., tumor necrosis factor-alpha and interferon-gamma), which promote endothelial dysfunction and vascular remodeling [20,21]. Furthermore, lymphocytes, particularly CD4+ T cells, have been found to contribute to increased PVR and the progression of PAH [9,10]. C-reactive protein is an acute-phase reactive protein mainly produced by the liver in response to inflammation and is closely related to the pathogenesis and prognosis of PAH [22,23]. Specifically, elevated CRP levels in patients with PAH are associated with an increased risk of disease severity and clinical deterioration [22]. CRP may contribute to endothelial dysfunction, inflammation, and vasoconstriction in the pulmonary arteries, thereby exacerbating the pathology of PAH [24]. Our study revealed that the ratio of lymphocytes to CRP had a good predictive value for the prognosis of patients with PAH. The lower the ratio of lymphocytes to CRP, the worse the prognosis of patients with PAH, which may be associated with the weakened immunity and the presence of inflammation [25,26,27]. The results of our study suggest that the LCR may reflect the balance between immune response and inflammation; however, further research is needed to validate the specific mechanisms underlying the LCR in patients with PAH.

Our study confirmed the importance of inflammatory parameters in the risk stratification of PAH. The variables of WHO-FC, 6MWD, and BNP/NT-proBNP are included in the risk stratification of PAH prognosis according to the 2015 and 2022 European Society of Cardiology and the European Respiratory Society Guidelines [2,28]. Similarly, WHO-FC, 6MWD, and BNP are also incorporated into the REVEAL and COMPERA risk assessment strategies [29,30,31]. The PAH subtype is also listed in the REVEAL risk score [29,31]. Consistent with the above studies, our study included PAH subtype, WHO-FC, 6MWD, and BNP as a clinical model for predicting the prognosis of PAH. Intriguingly, the predictive abilities of both the clinical model and the ESC model have been significantly improved after the addition of the LCR, indicating that inflammation has great potential to improve the risk stratification of PAH.

This study has several limitations that warrant consideration. Firstly, the sample size of patients with PAH is relatively small and sourced from a single center, which may affect the stability of our results and restrict the generalizability of our findings. Secondly, the retrospective nature of our study introduces potential biases, including selection bias and information bias, which could compromise the reliability of our conclusions. Thirdly, due to the nature of retrospective analysis and the complexity of clinical situations, we were unable to assess the impact of variations in the LCR on the prognosis of PAH, which may be influenced by corticosteroids or other drugs during the follow-up period. Lastly, although the LCR may reflect the balance between immune response and inflammation in patients with PAH, our study cannot accurately address the potential mechanisms of the LCR in the prognosis of PAH.

## 5. Conclusions

In conclusion, our study underscores the prognostic significance of the lymphocyte-to-C-reactive protein ratio in patients with PAH. The findings suggest that the LCR is a valuable predictive marker for clinical worsening events in patients with PAH. The nomogram based on the PAH subtype, 6MWD, WHO-FC, and LCR demonstrates good performance in predicting the adverse outcomes of PAH, providing an easily accessible and cost-effective tool for clinicians to make better clinical decisions.

## Figures and Tables

**Table 1:** Baseline characteristics of patients with pulmonary arterial hypertension.

Characteristics	Total (*n* = 116)	Non-Events (*n* = 49)	Events * (*n* = 67)	*p* Value
Age (year)	41.53 (13.33)	39.92 (12.75)	42.70 (13.71)	0.268
Female (%)	107 (92.2)	47 (95.9)	60 (89.6)	0.206
Height (cm)	160.00 (7.57)	160.45 (7.75)	159.67 (7.48)	0.587
Weight (kg)	55.82 (11.57)	56.17 (12.72)	55.56 (10.74)	0.779
BMI (kg/m^2^)	21.60 (3.40)	21.68 (3.81)	21.55 (3.10)	0.838
6MWD (m)	382.49 (121.58)	412.96 (122.51)	360.21 (116.83)	0.020
PAH subtype				0.224
IPAH	28 (24.1)	8 (16.3)	20 (29.9)	
CTD-PAH	66 (56.9)	32 (65.3)	34 (50.7)	
CHD-PAH	17 (14.7)	8 (16.3)	9 (13.4)	
PoPH	5 (4.3)	1 (2.0)	4 (6.0)	
WHO-FC (%)				0.023
Ⅰ/Ⅱ	79 (68.1)	39 (79.6)	40 (59.7)	
Ⅲ/Ⅳ	37 (31.9)	10 (20.4)	27 (40.3)	
Initial therapy (%)				0.506
Monotherapy	75 (64.7)	31 (63.3)	44 (65.7)	
Dual therapy	36 (31.0)	15 (30.6)	21 (31.3)	
Triple therapy	5 (4.3)	3 (6.1)	2 (3.0)	
Right Heart Catheterization				
RAP (mm Hg)	9.36 (4.74)	9.42 (4.44)	9.32 (4.98)	0.911
mPAP (mmHg)	51.19 (13.05)	52.65 (13.33)	50.12 (12.84)	0.304
PAWP (mmHg)	11.11 (3.56)	11.43 (3.88)	10.89 (3.32)	0.419
CO (L/min)	4.29 (1.55)	4.30 (1.48)	4.29 (1.62)	0.987
Cardiac index (L/min/m^2^)	2.74 (0.96)	2.74 (0.91)	2.74 (1.01)	0.996
PVR (Wood units)	11.31 (6.31)	11.66 (6.68)	11.05 (6.06)	0.605
SvO_2_ (%)	66.93 (10.26)	67.23 (9.64)	66.71 (10.75)	0.790
Echocardiography				
RV Diameter (mm)	45.67 (6.98)	46.22 (6.62)	45.27 (7.26)	0.474
TAPSE (mm)	16.29 (3.06)	16.23 (2.96)	16.33 (3.15)	0.867
PASP (mmHg)	77.50 (24.52)	76.78 (21.75)	78.03 (26.52)	0.787
TAPSE/PASP	0.24 (0.13)	0.23 (0.08)	0.25 (0.16)	0.459
Laboratory parameters				
CRP (mg/L)	3.57 (1.30–7.02)	2.97 (0.80–5.90)	4.62 (2.50–7.68)	0.027
WBC (10^9^/L)	6.44 (2.19)	6.32 (2.25)	6.52 (2.16)	0.623
Lymphocyte (10^9^/L)	1.68 (0.72)	1.62 (0.64)	1.73 (0.78)	0.395
Monocyte (10^9^/L)	0.38 (0.30–0.50)	0.38 (0.30–0.50)	0.39 (0.30–0.50)	0.971
Neutrophil (10^9^/L)	4.25 (1.89)	4.20 (1.96)	4.29 (1.85)	0.813
Platelet (10^9^/L)	189.72 (75.73)	208.57 (72.01)	175.94 (75.93)	0.021
BNP (pg/mL)	359.19 (547.08)	371.37 (588.94)	350.28 (518.70)	0.839
LCR	1.10 (1.63)	1.57 (2.14)	0.75 (1.01)	0.007

* Events were defined as the composite endpoint of clinical worsening events, including all-cause death, lung transplantation, and rehospitalization for PAH. Data are shown as *n* (%), mean (standard deviation), or median (interquartile range). Abbreviations: BMI, body mass index; 6MWD, 6 min walk distance; PAH, pulmonary arterial hypertension; IPAH, idiopathic PAH; CTD-PAH, connective tissue disease-associated PAH; CHD-PAH, congenital heart disease-associated PAH; PoPH, portopulmonary hypertension; WHO-FC, WHO cardiac functional class; RAP, right atrial pressure; mPAP, mean pulmonary artery pressure; PAWP, pulmonary artery wedge pressure; CO, cardiac output; PVR, pulmonary vascular resistance; SvO_2_, mixed venous oxygen saturation; RV, right ventricle; TAPSE, tricuspid annular plane systolic excursion; PASP, pulmonary artery systolic pressure; CRP, C-reactive protein; WBC, white blood cell; BNP, brain natriuretic peptide; LCR, lymphocyte-to-C-reactive protein ratio.

**Table 2:** Multivariate Cox proportional hazards analysis of LCR for clinical worsening events in patients with PAH.

Variables	Hazard Ratio (95% Confidence Interval)					
	Model 1	*p* Value	Model 2	*p* Value	Model 3	*p* Value
1-year						
LCR, Continuous	0.612 (0.361–1.037)	0.068	0.636 (0.374–1.081)	0.095	0.631 (0.318–1.254)	0.189
LCR, Category	0.357 (0.107–1.188)	0.093	0.383 (0.114–1.285)	0.120	0.271 (0.057–1.280)	0.099
3-year						
LCR, Continuous	0.688 (0.515–0.918)	0.011	0.708 (0.531–0.944)	0.019	0.708 (0.531–0.944)	0.019
LCR, Category	0.283 (0.121–0.660)	0.004	0.299 (0.128–0.698)	0.005	0.299 (0.128–0.698)	0.005
5-year						
LCR, Continuous	0.744 (0.594–0.933)	0.010	0.760 (0.606–0.954)	0.018	0.772 (0.614–0.970)	0.026
LCR, Category	0.343 (0.169–0.693)	0.003	0.356 (0.176–0.722)	0.004	0.361 (0.178–0.732)	0.005

Model 1: no adjustment; model 2: adjusted for age, sex, and WHO-FC; Model 3: adjusted for model 2 plus BMI, 6MWD, initial therapy, PAH subtype, BNP, neutrophil, monocyte, RAP, mPAP, PAWP, PVR, SvO_2_, and TAPSE/PASP. Abbreviations: LCR, lymphocyte-to-C-reactive protein ratio; PAH, pulmonary arterial hypertension.

## Data Availability

The data can be obtained from the corresponding author upon requirement.

## Abbreviations

6MWD	6 min walk distance
AUC	Areas under the ROC curve
BMI	Body mass index
BNP	Brain natriuretic peptide
CHD-PAH	Congenital heart disease-associated PAH
CI	Confidence interval
CO	Cardiac output
CRP	C-reactive protein
CTD-PAH	Connective tissue disease-associated PAH
HR	Hazard Ratio
IPAH	Idiopathic PAH
LASSO	Least absolute shrinkage and selection operator
LCR	Lymphocyte-to-C-reactive protein ratio
mPAP	Mean pulmonary artery pressure
PAH	Pulmonary arterial hypertension
PASP	Pulmonary artery systolic pressure
PAWP	Pulmonary artery wedge pressure
PoPH	Portopulmonary hypertension
PVR	Pulmonary vascular resistance
RAP	Right atrial pressure
ROC	Receiver operating characteristic
RV	Right ventricle
SvO_2_	Mixed venous oxygen saturation
TAPSE	Tricuspid annular plane systolic excursion
WBC	White blood cell
WHO-FC	WHO cardiac functional class
